# Boron and Coumaphos Residues in Hive Materials Following Treatments for the Control of *Aethina tumida* Murray

**DOI:** 10.1371/journal.pone.0153551

**Published:** 2016-04-19

**Authors:** Cesar Valdovinos-Flores, Octavio Gaspar-Ramírez, María Elena Heras–Ramírez, Carlos Lara-Álvarez, José Antonio Dorantes-Ugalde, Luz María Saldaña-Loza

**Affiliations:** 1 Departamento de Medicina Genómica y Toxicología Ambiental/ Instituto de Investigaciones Biomédicas/ Universidad Nacional Autónoma de México, Ciudad de México, México; 2 Centro de Investigación y Asistencia en Tecnología y Diseño del Estado de Jalisco, Unidad Noroeste Apodaca, Nuevo León, México; 3 Centro de Investigación en Matemáticas, Unidad Zacatecas, Zacatecas, México; 4 Servicios Apícolas de Querétaro, Querétaro, México; Ghent University, BELGIUM

## Abstract

In the search of alternatives for controlling *Aethina tumida* Murray, we recently proposed the BAA trap which uses boric acid and an attractant which mimics the process of fermentation caused by *Kodamaea ohmeri* in the hive. This yeast is excreted in the feces of *A. tumida* causing the fermentation of pollen and honey of infested hives and releasing compounds that function as aggregation pheromones to *A. tumida*. Since the boron is the toxic element in boric acid, the aim of this article is to assess the amount of boron residues in honey and beeswax from hives treated with the BAA trap. For this aim, the amount of bioaccumulated boron in products of untreated hives was first determined and then compared with the amount of boron of products from hives treated with the BAA trap in two distinct climatic and soil conditions. The study was conducted in the cities of Padilla, Tamaulipas, and Valladolid, Yucatan (Mexico) from August 2014 to March 2015. The quantity of boron in honey was significantly less in Yucatan than in Tamaulipas; this agrees with the boron deficiency among Luvisol and Leptosol soils found in Yucatan compared to the Vertisol soil found in Tamaulipas. In fact, the honey from Yucatan has lower boron levels than those reported in the literature. The BAA treatment was applied for four months, results show that the BAA trap does not have any residual effect in either honey or wax; i.e., there is no significant difference in boron content before and after treatment. On the other hand, the organophosphate pesticide coumaphos was found in 100% of wax samples and in 64% of honey samples collected from Yucatan. The concentration of coumaphos in honey ranges from 0.005 to 0.040 mg/kg, which are below Maximum Residue Limit (MRL) allowed in the European Union (0.1 mg/kg) but 7.14% of samples exceeded the MRL allowed in Canada (0.02 mg/kg).

## Introduction

Mexican honey is very important both nationally and internationally. In 2013, Mexico had 1 933 105 hives, which produced 56 907 tons of honey [1]. In particular, the states of Yucatan, Campeche and Quintana Roo produce 40% of honey produced in Mexico [1] and they export honey to Germany, Spain, Switzerland, Italy, France, United States, Canada, Saudi Arabia, Belgium and recently to Portugal, Colombia, and Panama [2]. The vast majority of producers in the Yucatan peninsula are micro- and small-scale indigenous farmers [3].

The arrival of the small hive beetle (SHB), *Aethina tumida* Murray, in Mexico represents a challenge for beekeepers and government institutions. SHB is native to sub-Saharan Africa, where it does not cause economic problems; but it can cause great harm to vulnerable haplotypes of honey bees. Its distribution has spread to other continents due to the marketing of food products which offer conditions for transporting specimens of SHB.

The first report of SHB in America was in 1996 in Charleston, South Carolina [4]. In 2000 it was reported in Egypt, in 2008 in Australia [5] and recently (2014) it has arrived to Europe [6]. In Mexico, SHB was officially first detected in Coahuila in 2007, and it has been reported in the states of Nuevo Leon, Guanajuato, Michoacan, San Luis Potosi, Tamaulipas, Quintana Roo, Yucatan, and recently, in Jalisco, Coahuila and Campeche [3].

*A. tumida* belongs to the Nitidulidae family, which contains 172 genera and about 2800 species [7]. This beetle lives and reproduces inside the hives of bees, feed on pollen, brood and bees-waste dropped to the floor of the hive [3, 4]. The larvae do not ferment honey –their activity leads to honey fermentation because of yeast carried on its body [8]. *A. tumida* often uses cryptic low-level reproduction [9] and it is photophobic [3]; then, small populations are hard to detect.

### Boric Acid and Attractant Trap

It is important to explore safe alternatives to control *A. tumida*. In [10], the authors proposed the BAA trap (Boric Acid with Attractant) which induces the death of 90% ± 10% of beetles in seven days under conditions of *ad libitum* access to food. The boric acid is an inorganic, water-soluble and slightly toxic pesticide for humans (toxicological classification IV [11]). BA is permitted for urban, domestic and agricultural use for the control of cockroaches, ants, silverfish, termites, scorpions, spiders and beetles [11]; its mechanism of action has not been clearly established [12, 13].

**Fig 1:**
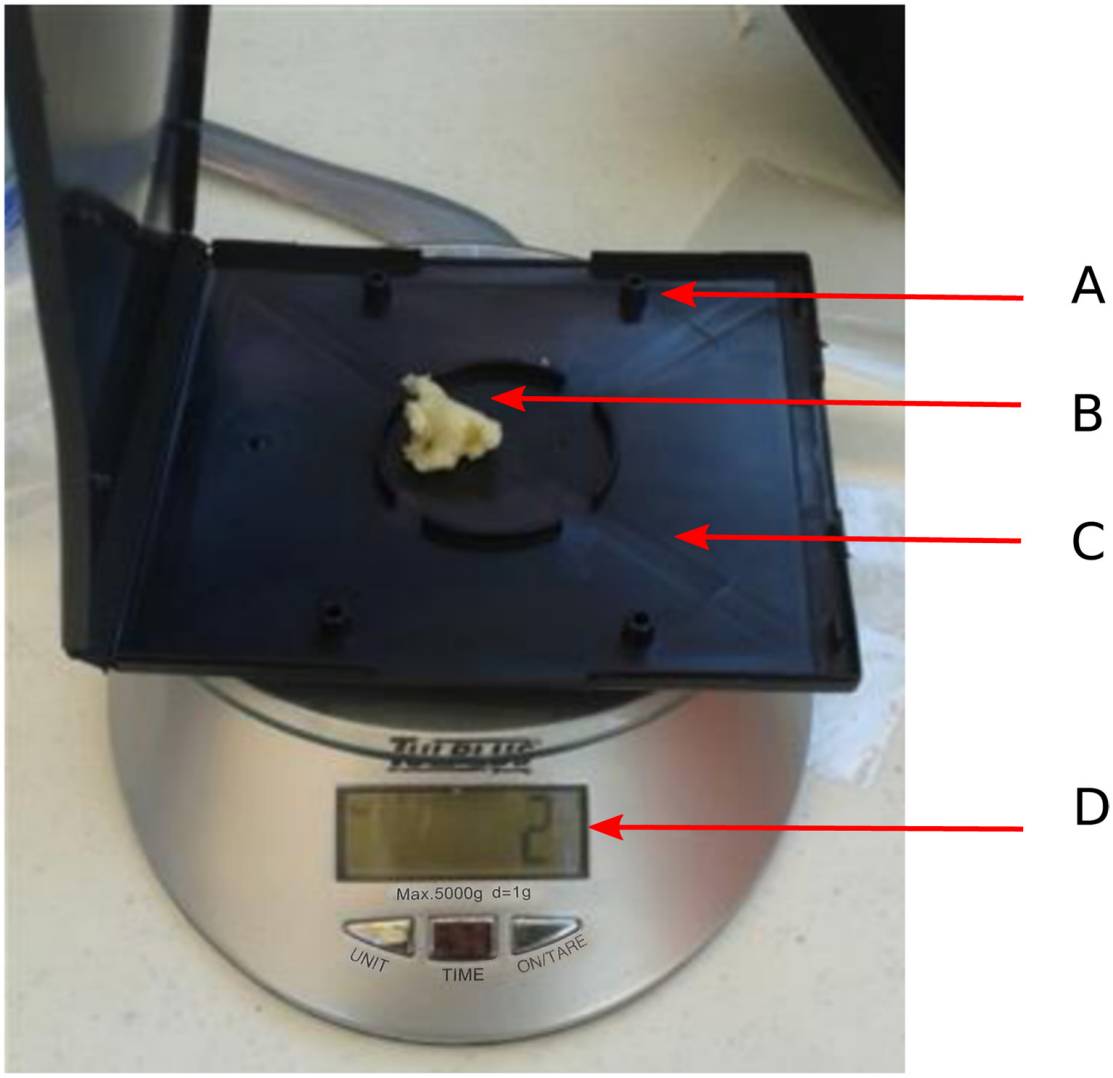
BAA Trap. (a) trap case: black to offer protection against light, dimensions 125 × 143 × 12 mm and with windows of 3.0 × 40.0 mm to prevent access by bees, (b) prepared bait, (b) four snaps for sealing, and (d) scale.

As shown in Fig 1, the BAA trap looks like a conventional CD case with windows of 3.0 × 40.0 mm allowing the pass of specimens of *A. tumida* but preventing access by bees. The trap is reinforced by four snaps for sealing, and it is black for offering a place against the light. The 2g of the bait placed inside the trap contains: 50% boric acid (Searles Valley Minerals), 17% shortening (INCA, ACH Foods, Mexico), 0.5% live yeast (Lessafrer, Saccharomyces cerevisiae [Meyen ex EC Hansen]), 0.5% sugar, and 32% of chopped fresh pineapple chunks. The attractant is very important in the BAA trap and it was chosen according to the following criteria [10]: (i) *A. tumida* usually eats hive food sources but it can also survive with alternative food such as mango, banana, grapes, avocado, pineapple, melon, and star fruit [14, 15]; and (ii) there is a mutual relationship between *A. tumida* and the yeast *Kodamaea ohmeri* lodged in the digestive tract. This yeast is excreted in the feces of the insect causing fermentation of pollen and honey in infested hives. The fermentation releases compounds that function as aggregation pheromones to *A. tumida*[16]. The fermentation process attracts beetles, thus populations are concentrated in areas with higher food availability for optimizing their reproduction.

Boron has a low toxicity in humans. But, side effects have been reported from high dosages of boron; e.g. it can affect the male reproductive system and fetal development [12, 13]. The BAA trap was proposed because boric acid is essentially non-toxic to honey bees [12, 17]. In addition, although high concentrations of boric acid can be toxic to bees, it has been demonstrated that application of boron fertilizer to crops did not affect the concentration of boron in honey [17]. Nevertheless, the trap is placed inside the hive and the maxim expresses that “*Sola dosis facit venenum*” (it is only the dose that makes a drug a poison); hence, it is important to assess the amount of boron residues in honey and beeswax from hives treated with the BAA trap.

### Coumaphos

The organophosphate coumaphos (toxicological classification II) is an insecticide and acaricide, lipophilic, and highly persistent (more than a year in soil) [11, 18]. It can be used against SHB; but, in many countries it is not authorized for treating bees [11]. Despite this, residues of coumaphos are usually detected in bee products [19]. Pesticide misuse –e.g., the application of unregistered formulations or pesticides intended for other uses.– may leave some of these residues. Such is the case of phenylpyrazole fipronil, pyrethroid deltamethrin, and in particular a mixture known in the Yucatan Peninsula as ‘magic dust’. This dust (powder) is an unlabeled formulation obtained from the illegal market that some beekeepers sprinkle inside the hive. In the previous work [3, 10], a reduced number of beetles was found in hives of Yucatan precluding mortality field studies of the BAA trap, mostly likely due to the presence of ‘magic dust’ in the hives. Hence, the so-called ‘magic dust’ and bee products were also analyzed for determining their coumaphos content.

## Materials and Methods

### Study area

Tests were carried out in the neighborhoods of Padilla, Tamaulipas, and Valladolid, Yucatan, Mexico in the period from August 2014 to March 2015. These locations reported the presence of SHB and were selected to compare residues in hives under different climatic conditions (i.e., temperature and precipitation).

**Fig 2:**
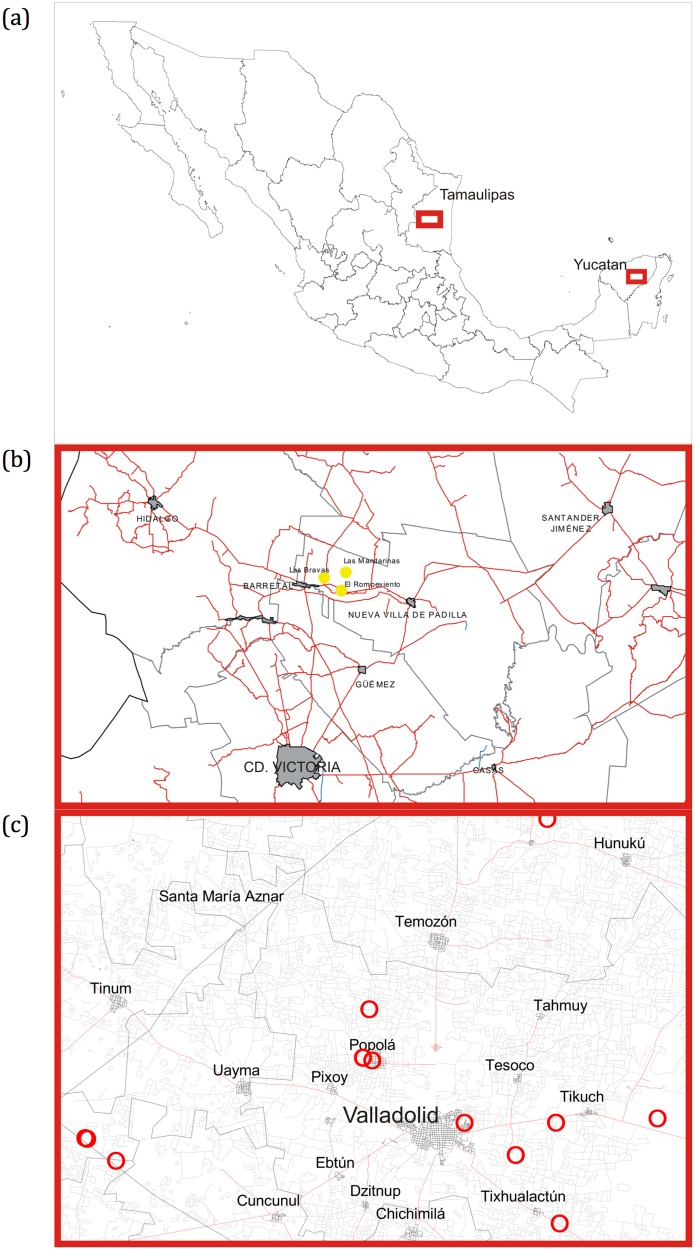
Geographic location. (a) Map of Mexico showing the two sampling sites. (b) Sampling sites (filled circles) in Padilla, Tamaulipas, Mexico. (c) Sampling sites (red circles) in Yucatan, Mexico.

The area near Padilla, Tamaulipas, Mexico is located between parallels 24°02’ and 24°07’ North latitude, and between 99°01’ and 99°05’ West longitude, at an altitude around 180 meters. It is very warm and warm dry with summer rains, the temperature ranging between 1 and 43°C, the annual rainfall is 700 mm in average, and the soil type is predominantly Vertisol [20] (Fig 2b).

The area around Valladolid, Yucatan, Mexico is located between parallels 20°24’ and 20°54’ North latitude; meridians 87°57’ and 88°21’ West longitude, at an altitude between 10 and 30 meters. It is warm humid with rains in summer, with an average annual temperature of 26°C, and a maximum of 36°C and a minimum of 16°C. The total annual rainfall is between 1100 and 1500 mm. Its surface is limestone sedimentary rock, with Leptosol and Luvisol soils [21] (Fig 2c).

### Treatment, collection and sample preparation

All field work was conducted in privately-owned apiaries and with permission of the beekeepers and the land owners. No endangered or threatened species were involved in the study.

The study included 16 hives from three apiaries of Tamaulipas, and 14 hives from 14 apiaries of Yucatan. Hives of each region were randomly divided into two groups; the following treatments were applied:

**Group A.** This group of hives was used as control; hence, no treatment was applied, and

**Group B.** This group was treated with the BAA trap for 4 months, with bait replacement every eight days as explained in [10].

In Yucatan only post-treatment samples were taken, while in Tamaulipas pre-and post-treatment samples were taken for comparison over the time. Each bioassay was performed with at least five replicates in accordance with the recommendation of the preliminary Mexican Official Standard NOM-000-SAG / ZOO-2014 [22]. Thereafter, 150g of beeswax and 150g of honey were sampled from each experimental hive. Samples were collected in plastic tubes and sent on ice to CIATEJ (Centro de Investigación y Asistencia en Tecnología y Diseño del Estado de Jalisco, México) for analysis.

### Determination of residues

The boron content in samples was determined by atomic absorption spectrometry, according to the method established in the NOM-117-SSA1-1994 [23].

To determine the residue level of coumaphos in each sample, the following equipment was used: a liquid chromatograph (Agilent Technologies, model 1260 and 1290 Infinity) coupled to a quadrupole–time-of-flight mass spectrometer (G6530A), and autosampler with FLUKA 1260 (Sigma-Aldrich St. Louis MO, USA). The coumaphos extraction from samples of honey and wax was carried out according to the method QuEChERsr Agilen Technologies (Santa Clara, CA, USA) [24], which is the official method of the AOAC 2007.01 [25]. Samples of ‘magic dust’ were also analyzed for coumaphos and were diluted in acetonitrile. Triphenylphosphate (Sigma-Aldrich-Supelco, Bellefonte, PA, USA) was used as internal standard.

### Statistical analysis

Data are represented as mean ± S.E. and the significance was assessed by Student’s t test for paired data from Tamaulipas and unpaired data for Yucatan. The Mann–Whitney U test was used to compare the boron content between regions; for this case, data are represented as median ± interquartile range. All data (Table in S1 Dataset) were analyzed using the statistical package SigmaPlot^®^11(Systat Software Inc.). P values less than 0.05 were considered statistically significant.

## Results

**Boron levels in honey and wax.** Samples collected in Tamaulipas before and after treatment did not show any significant difference in the boron level (Table 1), either in samples from control hives (p = 0.16 for honey and p = 0.23 for wax) or in samples from treated hives (p = 0.32 for honey and p = 0.89 for wax). There was also no statistical difference in boron levels between treated and untreated samples collected from Yucatan (p = 0.48 for honey and p = 0.27 for wax), as shown in Table 2,.

**Table 1:** Pre-and post-treatment levels of boron in samples collected from Tamaulipas. Data are presented as mean ± SE (mg/kg).

	pre-treatment	post-treatment	p value
Honey			
control	6.44 ± 0.04	7.47 ± 0.57	0.16
BAA	6.11 ± 0.35	6.63 ± 0.36	0.32
Beeswax			
control	8.96 ± 1.44	11.55 ± 1.50	0.23
BAA	11.70 ± 3.55	11.09 ± 2.65	0.89

**Table 2:** Comparison of boron levels between treated and untreated groups in samples collected from Yucatan. Data are presented as mean ± SE (mg/kg).

	control	BAA	p value
Honey	4.68 ± 0.37	5.09 ± 0.43	0.48
Beeswax	19.71 ± 4.40	13.76 ± 3.80	0.27

**Fig 3:**
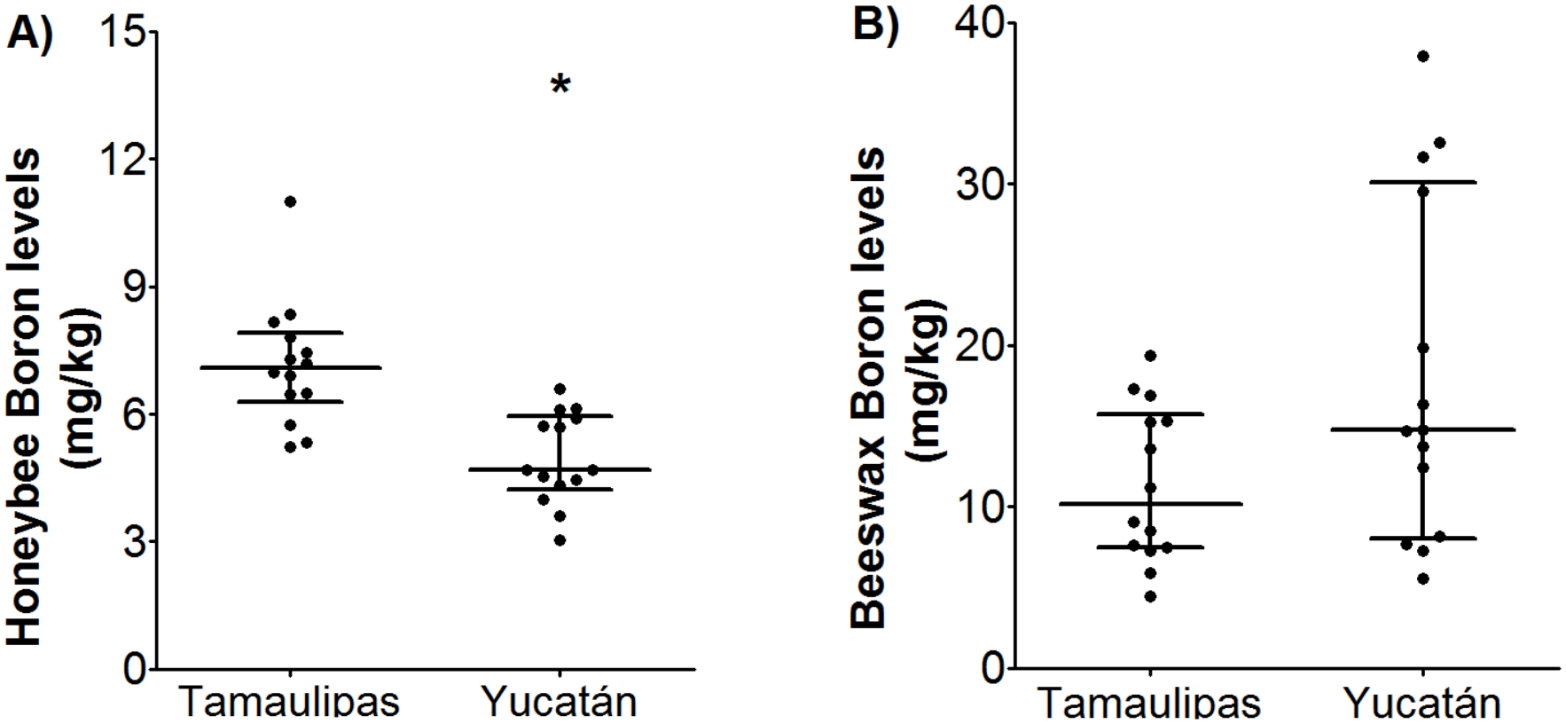
Comparison of boron levels of hives in Yucatan against hives in Tamaulipas. (A) honey, and (B) beeswax. Each point is a sample; the middle bar represents the median ± interquartile range (n = 14). Data were analyzed using Mann Whitney U test * p = 0.01.

On the contrary, results show a significant difference in boron content in honey between the two studied regions (Mann Whitney p = 0.001); the concentration of boron in honey collected from Tamaulipas was higher (6.071 ± 1.53 mg/kg) than the concentration found in Yucatan (4.89 ± 1.11 mg/kg) as shown in Fig 3. Furthermore, a great variation was observed in the boron content in wax 12.871 ± 8.047 mg/kg as shown in Fig 3.

**Coumaphos levels.** The ‘magic dust’ has coumaphos in concentrations that range from 639.55 to 900 mg/kg (Table 3). This pesticide was found in 100% of wax samples in concentrations ranging from 0.155 to 2.220 mg/kg; moreover, it was also found in 64% of the honey samples in concentrations that range from 0.005 to 0.040 mg/kg.

**Table 3:** Proportion of samples with coumaphos residues detected above the Limit of Quantification (LOQ) of 0.005; it also shows the concentration range, median and 75% percentile of total samples.

	Detections			Concentration (mg/kg)				
	N	Samples	%	Range	Mean	SE	Median	75%tile
Honey	9	14	64	0.005—0.040	0.012	0.0037	0.006	0.013
Beeswax	14	14	100	0.155–2.220	0.958	0.17	0.881	1.101
Magic dust	1	1	100	639.55–900	0.005	-	-	-

## Discussion

Although the bait of the BAA trap contains boric acid, the window size was calculated for preventing access by bees [3, 10]. Unlike most pesticides, boron is an element that is widely distributed in the earth’s crust, so inevitably organisms are exposed to it. Regardless of which boron compound is employed as a pesticide, at physiological pH all boron salts are found as boric acid and cannot be metabolized by organisms [12].

Therefore, the toxic effect of boric acid is similar to those containing boron compounds. Thus, the boron equivalents (Table 4) can be used for quantitatively comparing the effects of different boral compounds. Boron is present in soils in concentrations ranging in 10 to 300 mg/kg and 30 mg/kg on average depending on the type of soil, the amount of organic matter, and the amount of rainfall. In the surface water the boron is in the range 0.001 to 360 mg/l [12, 13].

**Table 4:** Conversion factors to equivalent doses of boron [13].

boron compounds	Formula	Conversion Factor
boric acid	H_3_BO_3_	0.175
sodium tetraborate	Na_2_B_4_O_7_ 10H_2_O	0.113
sodium octaborate	Na_2_B_8_O_11_ 4H_2_O	0.210
zinc borate	2ZnO 3B_2_O_3_ H_2_O	0.149

Normally, honey has boron coming from pollen and nectar of vascular plants [26]. Previous studies reported that the average boron content in honey was 7.2 mg/kg [27], 6.07 mg/kg [28] and 27.26 mg/kg [17]. These levels are comparable to those observed in honey from Tamaulipas (6.071 mg/kg), while honey from Yucatan contained a lower concentration of boron (4.89 mg/kg). Siede et al [17] showed that a low concentration of boron (10 mg/kg) does not affect bee survival and that the lethal concentration after continuous oral uptake is between 100 and 500 mg/kg. An increase of boron content was expected during the natural process of honey production [17], but in our study there were no significant changes neither in honey nor in wax.

Food is the primary source of intake for the non-occupationally exposed population [12, 13]; e.g., almonds, prunes, and raisins have boron concentrations of 23, 27, and 25 mg/kg, respectively [27]. According to the Environmental Protection Agency (EPA) the oral reference dose of boron for human consumption is 0.2 mg/kg per day [13], while for the World Health Organization (WHO) the tolerable intake of boron is 0.4 mg per kilogram of body weight per day [12].

The estimated consumption of honey is 21 g/day (for infants younger than one year) to 96 g/day (for adults over 50 years) [18]. Results show that the content of boron in honey from Tamaulipas and Yucatan is 6.071 ± 1.537(SD) mg/kg; it means that for the consumption estimated in [18], the boron consumed through honey would be in the range 0.13 to 0.58 mg/day, which is below MRL set by the EPA [12] and the WHO [13].

The level of boron in honey from treated hives of Yucatan (5.09 ± 0.43 mg/kg) is lower than that reported by literature because the soil in this area is rich in carbonates and high in pH, generating micronutrient deficiencies; consequently, it is common to find lower boron levels than the reported average [29]. This phenomenon also explains the difference of boron in honey between Tamaulipas and Yucatan (Fig 3). As far as the authors know, this is the first paper reporting the concentration of boron in wax, so there is no reference for comparison.

**Coumaphos levels.** Results show that the organophosphate pesticide coumaphos was in 100% of wax samples and in 64% of honey samples collected from Yucatan. Following the first discovery of *A. tumida* in the Yucatan peninsula, producers have used the so called ‘magic dust’ without knowing its composition or potential effects. The results from our analysis show that the primary active ingredient is the pesticide coumaphos at concentrations that range in 639.55 to 900 mg/kg (Table 3).

The impact of pesticides depends on their type of exposure –dust, aqueous solution, or adhesive strips– and exposure time [30]. There is growing evidence demonstrating the association between pesticide exposure and neurological disorders, as well as damages in infants exposed to pesticides even at concentrations that do not produce adverse effects to the mother [31, 32].

Since 1999, coumaphos has been approved by the U.S Environmental Protection Agency to control varroa mites and small hive beetles [18]; however, it is highly persistent [18].

In Mexico, it is allowed for the control of flies, ticks, lice, fleas, and mites on birds, cattle, sheep, goats, horses, canidae, and pigs, but it is not allowed to be used in honeybee hives [11]. The Maximum Residue Limit (MRL) in honey allowed by the legislation of the United States is 0.15 mg/kg, while the MRL in beeswax is 45 mg/kg [18]. In the European Union, coumaphos is not allowed for beekeeping use but it tolerates a MRL of 0.1 mg/kg of coumaphos in honey [33, 34]. Finally, Canada’s laws allow a MRL of 0.02 mg/kg in honey and 0.1 mg/kg in beeswax as honeycomb [35]. Our results show that 100% of wax samples and 7.14% of honey samples analyzed exceeded the MRL established in Canada. On the other hand, the level of coumaphos in honey was less than 0.040 mg/kg, meaning that honey from the study area meets MRL requirements of both the United States and the European Union.

Recent studies have shown that coumaphos in honeycomb wax affects bees during their development. The LC_50_ for *Apis mellifera* is 46.3 mg/L but in the larval stages it reduces to 8 mg/L [36]. Coumaphos strips in brood chamber with the queen induce sub lethal effects such as physical abnormalities, atypical behaviors, lower weight and lower weight ovaries of queen bees respect to controls [30]. Moreover, exposure to coumaphos increases offspring mortality [37].

## Conclusions

The BAA trap was inspired by the feeding and behavioral patterns of *A. tumida*. The in-hive trap uses boric acid as the insecticide and lead to 90% mortality of the beetles in seven days under controlled conditions with *ad libitum* food supply. In this research the residual effect of the BAA trap was studied and our results show no statistical difference in the boron content in honey collected from hives in the treated and control groups. These findings open the possibility of implementing this control method against *A. tumida*. This is the first paper that determines the levels of boron in beeswax.

The pesticide coumaphos is highly concentrated in the formulation known as ‘magic dust’, and is one of its active ingredients. The indiscriminate use of highly persistent insecticides such as coumaphos endanger the health of beekeepers, bees and consumers; especially when it is not regulated or when the producers have not been trained for its proper use.

The honey from the study areas meets the MRL requirements of both the United States and the European Union. But if beekeepers keep using ‘magic dust’, it is likely that coumaphos levels will rise. In such case, Mexican bee products could exceed the MRL allowed in many countries; now, a few honey samples of this study exceed the MRL of coumaphos in honey set in Canada

Research on pesticides of low toxicity, such as boric acid used by the BAA trap, is vital for this important activity. Therefore, the research of alternative methods against this pest must be continued in order to prevent beekeepers to use pesticides not allowed in honey bee hives as they have harmful effects on bees, consumers and beekeepers.

## Supporting Information

S1 Dataset Raw data of boron and coumaphos residues in honey and beeswax. Excel^®^ spreadsheet with data entries organized by location (Yucatan and Tamaulipas).
(XLSX)
